# Effect of the Geometry of Thin-Walled Aluminium Alloy Elements on Their Deformations after Milling

**DOI:** 10.3390/ma15249049

**Published:** 2022-12-18

**Authors:** Magdalena Zawada-Michałowska, Józef Kuczmaszewski, Paweł Pieśko

**Affiliations:** Department of Production Engineering, Faculty of Mechanical Engineering, Lublin University of Technology, Nadbystrzycka 38D, 20-618 Lublin, Poland

**Keywords:** aluminium alloy, deformation, thin-walled element, milling, aviation

## Abstract

The aim of this paper is to analyse the effect of the selected geometric properties of thin-walled structures on post-machining deformations. In the study, EN AW-7075 T651 and EN AW-6082 T651 aluminium alloys were used to prepare specially designed thin-walled sample elements, i.e., elements with walls arranged in a semi-open and closed structure and with a dimension of 165 × 262 × 50.8 mm consisting of bottom and vertical stiffening walls and so-called ribs with a thickness of 1 mm. The measurements of the absolute deformations of the thin-walled bottom were performed with the use of a Vista coordinate-measuring machine by Zeiss with a PH10 head by Renishaw. Based on the obtained results, it was found that absolute deformation values were higher for walls arranged in a semi-open structure. It is related to a lower rigidity of the tested structure resulting from the lack of a stiffening wall, which is the so-called “rib”. Notwithstanding the geometry of the elements, greater absolute deformation values were recorded following conventional cutting methods. The use of high-speed cutting (HSC) provided positive outcomes in terms of minimising the deformation of thin-walled elements. Additionally, it was found that higher absolute deformations were obtained for EN AW-7075 T651 alloy.

## 1. Introduction

According to the Global Service Forecast (GSF) published by Airbus for 2022–2041, the demand for passenger traffic will continue to grow at the rate of 3.6% a year in the next 20 years, and the number of passengers is likely to double by 2041 and amount to approximately nine billion. Moreover, it is forecast that the downturn resulting from the global COVID-19 epidemic will subside in 2023. It is also estimated that due to the need to replace existing aircraft with newer and environmentally friendly models, the demand for passenger and freight aircrafts is likely to grow at a stable rate. It is expected that the demand for new aircrafts (both passenger and freight ones) will amount to a total of 40,000 new vehicles in the period in question. It should be stressed that new generation energy-saving aircrafts currently account for only 20% of the fleet, and it is assumed that their share will amount to 95% in 2041. It is mostly associated with the efforts to reduce the weight of vehicles, thus allowing energy and fuel savings. Moreover, the aviation industry is at the same time closely related to the arms industry, and defence expenditures are growing proportionally aircrafts relative to the escalation of potential threats to security. The aforementioned forecasts confirm the rapid growth of aviation and the need to refine and search for new solutions within the sphere of designing and manufacturing aircrafts [1].

As aircraft is subject to changeable loads during flights due to the performance of numerous complex manoeuvres, their durability, reliability, and further maintenance are essential. One of the requirements imposed on state-of-the-art aircraft is the reduction in their weight, which is advantageous for their flight properties and allows decreases in operational costs. Therefore, thin-walled elements have begun being widely used in aircraft designs, accompanied by multiple reinforcement elements, e.g., longerons and stringers allowing appropriate rigidity and strength for the entire structure [2,3]. The aviation industry currently uses thin-walled integral (or structural) elements made of light metal alloys, mainly aluminium alloys [4], which are characterised by a uniform structure and lower weight in relation to their dimensions and when compared with traditional parts [5]. It should be noted that such elements constitute a load-bearing structure with increased strength parameters, which also translates into the increased durability of an aircraft [6]. In article [7], it was noted that the advantage of using integral thin-walled elements was also a reduction in fuel consumption. They also allow a decrease in the number of joints, contributing to the minimisation of labour intensity and assembly costs [8]. However, taking into account the problems occurring during production, it is necessary to assure the continued refinement of structural and technological solutions. There is currently a trend for simplifying semi-finished products; hence, such structures are chiefly made of monolithically rolled plates of light metal alloys, such as aluminium, magnesium [9,10], or titanium [11], which have substantial technical significance and play a vital role in numerous industry sectors. In such cases, it is necessary to take into account the anisotropy of mechanical and structural properties in individual directions in relation to the rolling direction. Furthermore, large quantities of chips, accounting for over 95% of a given semi-finished product, imply the need to resort to high performance cutting and high speed cutting [12,13], which fit into the trend of reducing manufacturing costs via a faster removal of machining allowances than observed in traditional methods [14,15,16]. At the same time, taking into account the thin walls and dimensions of the manufactured elements, the application of, e.g., plastic working is practically impossible. Moreover, the use of high performance milling allows, i.e., higher surface quality and the elimination of grinding processes, but it causes, e.g., faster tool wear [17]. The solution is to use cutting fluids, but these have tendencies to minimise wet cutting, so the alternatives are minimum quantity cooling lubrication (MQCL) and minimum quantity lubrication (MQL) [18,19,20]. It should be mentioned that thin-walled elements are also used in the military, marine and automotive industries [21,22].

The efforts to minimise the weight of the produced parts and the accompanying technological difficulties related to, i.e., the machining of thin-walled elements are significant issues both from the scientific and practical points of views. Various sectors, in particular the aviation industry, currently struggle with the issue of unwanted deformations of thin-walled elements following the completed machining process, and the removal of the force holding a given workpiece [23,24,25]. The deformations may range from several micrometres in the case of small parts to several or even a dozen centimetres in large-sized parts [7]. It is related to the reduced rigidity due to the cutting force applied in the machining process. It is a complicated issue, and addressing this issue in a comprehensive way is difficult, as the process of the occurrence of post-machining deformations is complex and depends on multiple factors. One of the main causes includes internal stress generated at each stage of a given technological process [26]. Various methods are used in the machine industry to eliminate internal stress: for instance, vibration and thermal methods. These are, however, cost-intensive operations that extend the production time, and this is why measures are being undertaken to eliminate them. The large dimensions of integral structures are also major problems. In addition, depending on the material used, technological guidelines might vary, and the guidelines are related to different properties of individual alloys [27].

The increase in the rigidity of a structure and minimising post-machining deformations are also possible via the application of, i.a., the following [28,29]:

- Elements of increased thickness, which ultimately results in the growth of the aircraft weight;
- Composite materials, which involves the need to apply technologies that ensure the repeatability of the mechanical properties of the entire structure (they are an alternative to light metal alloys [30,31,32,33]);
- Additional stiffening walls, which are so-called “ribs.”

The best solution to minimise deformations is to add rigidity to the structure by adding stiffening walls, “ribs”, which would result in the optimum use of materials without any significant increase in manufacturing costs [28,29].

The authors of a papers [34,35,36], in addition to other possibilities for minimising the deformation of large-sized elements, mention so-called “mirror milling” operations, which involve a new technology consisting in the use of two heads that are placed precisely at two sides of a thin-walled element and shift their position in a synchronised manner. The task of the first head is to hold the rear part of a workpiece, while a cutting tool is placed on the other head.

Li and Zhu [37] presented possibilities for minimising errors in the machining of thin-walled elements, particularly deformations caused by cutting forces, which are considered in the optimisation of the tool’s path. To predict the occurrence of errors when milling thin-walled elements and for compensation purposes, the finite element method was applied in studies by [38]. The prediction of dimensional errors of thin-walled parts in milling, which mostly are caused by static deflections, is shown in [39]. In research studies conducted by [40], analyses were performed to examine the effect of machining parameters on surface roughness and on the deformation of thin-walled elements. It was noted that the depth of the cut has the greatest impact on deformations. Kang et al. [41] suggested a surface-error prediction model, which was experimentally verified in the course of five-axis milling. It has been shown that the modelling of deformations of thin-walled parts results in an acceptable prediction of dimension errors prior to expensive experimental cutting tests. The authors of paper [42] demonstrated a methodology for surface fittings based on isometric mapping, allowing a reduced number of cutting errors, applied on the adaptive machining of large and curved thin-walled elements. Wang et al. [43] demonstrated different milling strategies comprising the removal of “blocks” in various sequences, and they indicated the possibility for minimising deformations of a thin-walled object in finishing machining operations.

The materials used to manufacture thin-walled integral elements include mainly aluminium alloys intended for plastic working, which contain approx. 5–6% of additional alloying elements, demonstrating a relatively good machinability, and they are characterised by increased strength [44,45].

Based on the literature analysis, it was found that papers are mainly theoretical scientific considerations and numerical simulations predicting the size of the resulting deformations. However, there is a lack of application information and technological guidelines that would help solve important problems of the machine industry related to the occurrence of deformations of thin-walled elements after machining. Only in a few papers were attempts made during the experimental verification of the developed theoretical models and the search for the possibility of practical applications in the technology of manufacturing such elements.

The aim of this paper is to analyse the effect of the selected geometric properties of thin-walled elements on post-machining deformations and on their minimisation. The use of additional stiffening ribs would allow the improvement of structure rigidity and a reduction in its vulnerability to deformation at a relatively low cost. In addition, the influence of technological parameters and materials on the resulting deformations was studied.

## 2. Materials and Methods

**Figure 1:**
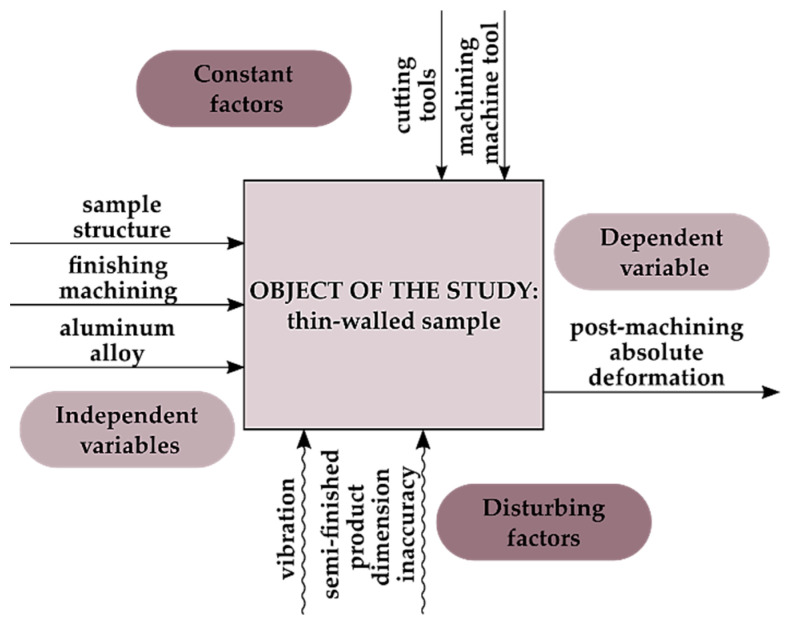
Study object model.

Figure 1 shows the study’s object model, which constitutes a thin-walled sample. Independent (input) variables included the sample’s structure, the finishing machining, and the workpiece material, while the dependent (output) variable included absolute deformations after cutting. Invariable factors included room conditions and the cutting machine. The confounding factors included the vibrations and inaccurate dimensions of semi-finished products. Aluminium alloys intended for plastic working were used in the study, such as EN AW-7075 T651 and EN AW-6082 T651, and two types of samples were prepared from the alloys, i.e., the semi-open structure (without the stiffening rib) and the closed structure (with a stiffening rib). Moreover, two sets of technological parameters were used for finishing machining, corresponding to conventional cutting and high speed cutting (HSC).

**Figure 2:**
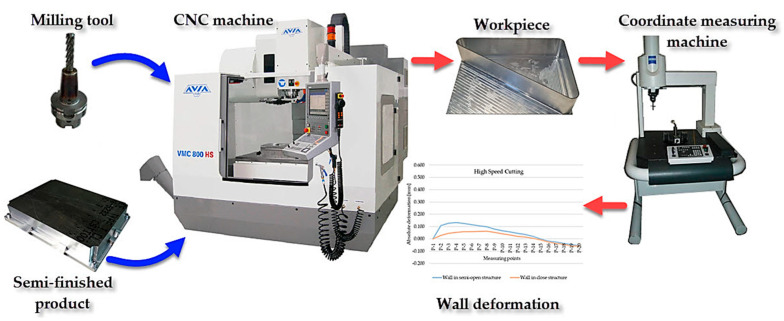
Study object model.

The experiment procedure plan is demonstrated in Figure 2. It takes into account the order of the activities, the research apparatus used, and the technical measures that are necessary for conducting the study.

The study is based on aluminium alloys EN AW-7075 T651 and EN AW-6082 T651, which are highly popular in the aviation industry and used in the manufacture of aircraft structure elements. Their chemical compositions are presented in Table 1 below.

In terms of machinability, the EN AW-7075 T651 aluminium alloy is classified in Group II. It is a material with good machinability that is characterised by the following mechanical properties [47]:

- Tensile strength: Rm min. = 525 MPa;
- Yield strength: *R_p_*_0,2_ min. = 440 MPa;
- Elongation: min. *A* = 4%;
- Hardness on the Brinell scale: 155 HB.

In relation to the EN AW-7075 T651 alloy, the EN AW-6082 T651 aluminium alloy (Group I) has weaker machinability, and it is characterised by the following mechanical properties [47]:

- Tensile strength: Rm min. = 295 MPa;
- Yield strength: *R_p_*_0,2_ min. = 240 MPa;
- Elongation: min. *A* = 8%;
- Hardness on the Brinell scale: 89 HB.

Both aluminium alloys for plastic working were subjected to heat treatments via precipitation hardening processes (T651 state). Based on a comparison of the mechanical properties of alloys EN AW-7075 T651 and EN AW-6082 T651, it was observed that the EN AW-7075 T651 alloy is characterised by greater strength and hardness, while the EN AW-6082 T651 alloy shows better ductility.

**Figure 3:**
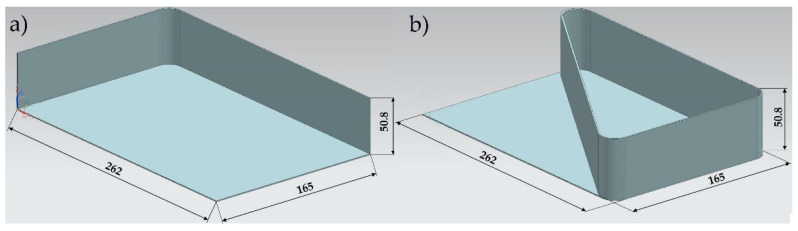
Thin-walled samples: (**a**) with a semi-open structure and (**b**) with a closed structure.

EN AW-7075 T651 and EN AW-6082 T651 aluminium alloys were used to prepare specially designed thin-walled samples, i.e., elements with walls arranged in a semi-open and closed structure with a dimension of 165 × 262 × 50.8 mm and consisting of a bottom and vertical stiffening walls and so-called ribs, with a thickness of 1 mm (Figure 3). Rolled plates with a thickness of 50.8 mm were used as semi-finished products. No pre-cutting operations were performed.

**Figure 4:**
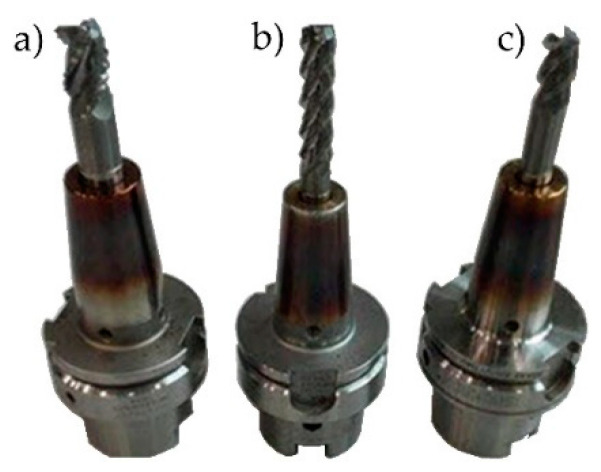
Machining tools: (**a**) SGS Tools 44303, (**b**) SGS Tools 44631, and (**c**) SEGER F103AL-120.

The cutting was performed on the Avia VMC 800HS vertical machining centre with high performance cutting and high speed cutting functions. With the aim of applying high speed cutting on aluminium alloys belonging to the ISO N material group, the following machining tools were applied (Table 2):

- SGS Tools 44303 (Wokingham, Berkshire, England): roughing of the bottom and the walls—high performance cutting (Figure 4a);
- SGS Tools 44631 (Wokingham, Berkshire, England): machining of the bottom and the vertical walls—conventional cutting and high speed cutting (HSC) (Figure 4b);
- SEGER F103AL-120 (Wiśniowa, Poland): used to “remove” the workpiece from the “frame” type fixture (Figure 4c).

**Table 2:** Technical data of the machining tools used (own elaboration, based on [49,50]).

Tool	SGS Tools 44303	SGS Tools 44631	SEGER F103AL-120
**Cutting diameter (mm)**	16	12	12
**Overall length (mm)**	92	100	81
**Length of cut (mm)**	32	48	24
**Number of flutes (-)**	3	4	3
**Helix angle (°)**	38	inconstant (38 to 41)	45

The SGS Tools 44631 cutter has a geometry dedicated for machining thin-walled elements, and it is characterised by changeable flute spacing and changeable helix angle. The tools were placed in HSK63A heat shrink toolholders, allowing the accurate mounting of a tool, and then they were balanced in class G2.5 at a rotational speed of 25,000 rpm in line with the standard [48] with the use of a CIMAT CMT 15 V2N balancing machine (Bydgoszcz, Poland).

**Figure 5:**
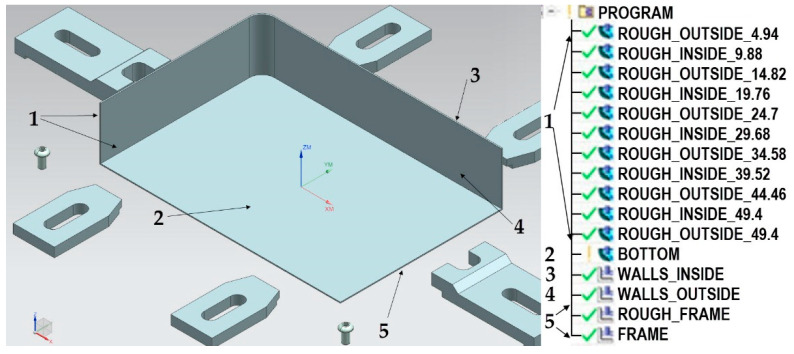
Stages of machining of the thin-walled element with walls arranged in a semi-open structure.

In both cases, the workpiece was fixed with the use of clamps and a “frame” fixture. The machining of the thin-walled element with walls arranged in a semi-open structure was performed in 5 stages (Figure 5):

1. Alternate roughing of outside and inside wall surface;
2. Machining of the bottom;
3. Finishing machining of the inside surface of walls;
4. Finishing machining of the outside surface of walls;
5. Cutting out of the structure from the frame.

**Figure 6:**
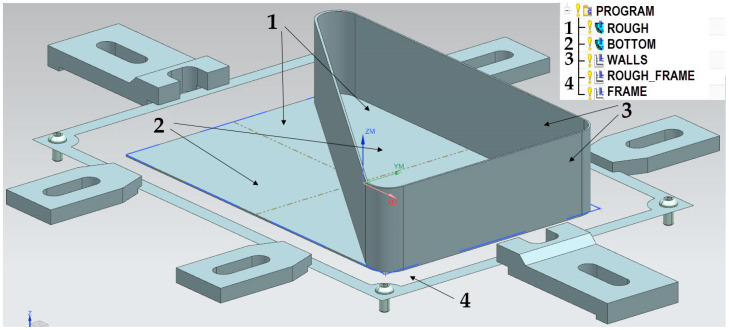
Stages of machining of the thin-walled element with walls arranged in a closed structure.

The machining of the thin-walled element with walls arranged in a closed structure was performed in the following stages (Figure 6):

1. Alternately roughing the inside and outside of the wall’s surface;
2. Machining the bottom;
3. Finishing machining of the inside and outside surfaces of walls;
4. Cutting out of the structure from the frame.

The roughing was performed with the use of technological parameters corresponding to high performance cutting, such as the following: *a_e_* = 12 mm; *a_p_* = 4.94 mm; *f_z_* = 0.13 mm/tooth; *v_c_* = 502 m/min. Allowances of 0.2 mm at each side of the walls and the bottom were left for finishing machining. Finishing machining was performed with two variant operations with the use of conventional cutting and HSC (Table 3).

The accuracy with respect to dimensions and shapes was assessed on the basis of the results from the absolute deformation of the thin-walled bottom with the use of a Vista coordinate-measuring machine by Zeiss (Zeiss, Oberkochen, Germany) with a PH10 head by Renishaw (Wotton-under-Edge, United Kingdom) equipped with a low-force TP-20 measuring probe and a special tip for measuring thin-walled elements.

**Figure 7:**
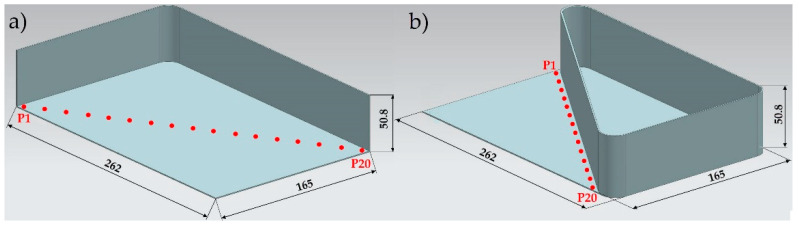
Absolute deformation measurements (**a**) of an element with walls arranged with a semi-open structure and (**b**) of an element with walls arranged with a closed structure.

The measurements were conducted along the diagonal line of the thin-walled samples under analysis, with results recorded from 20 measurement points (Figure 7).

## 3. Results

**Figure 8:**
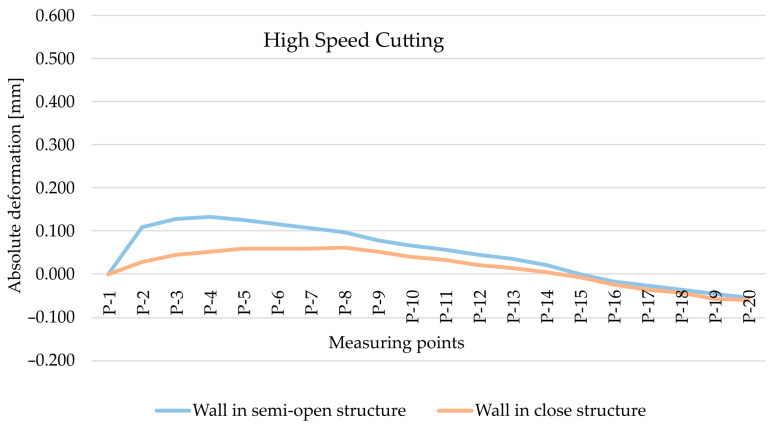
The comparison of absolute deformation for individual measurement points depending on the geometry of the thin-walled sample for HSC and for the EN AW-7075 T651 aluminium alloy.

**Figure 9:**
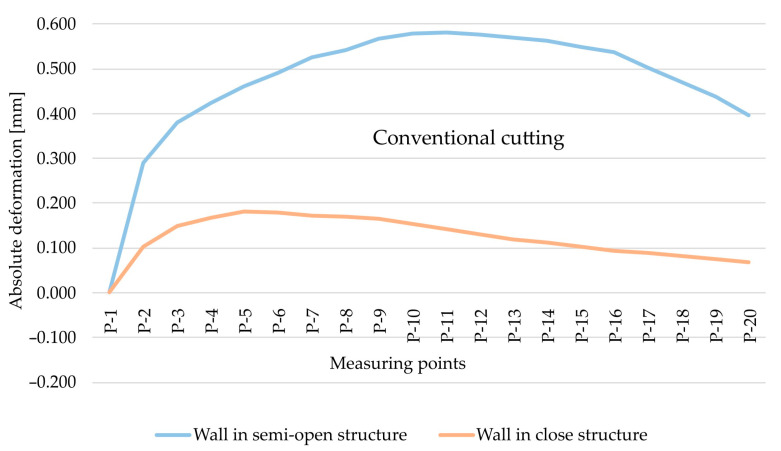
The comparison of absolute deformation for individual measurement points depending on the geometry of the thin-walled sample for conventional cutting and for the EN AW-7075 T651 aluminium alloy.

The analysis began with the results obtained for the EN AW-7075 T651 alloy and the comparison of absolute deformation values depending on the geometry of the thin-walled sample for individual measurement points, taking into account two cutting types, i.e., HSC (Figure 8) and conventional cutting (Figure 9). Based on the obtained results, it was found that, in both cases, absolute deformation values were higher for walls arranged in a semi-open structure. It is related to a lower rigidity of the tested element resulting from the lack of a stiffening wall.

**Figure 10:**
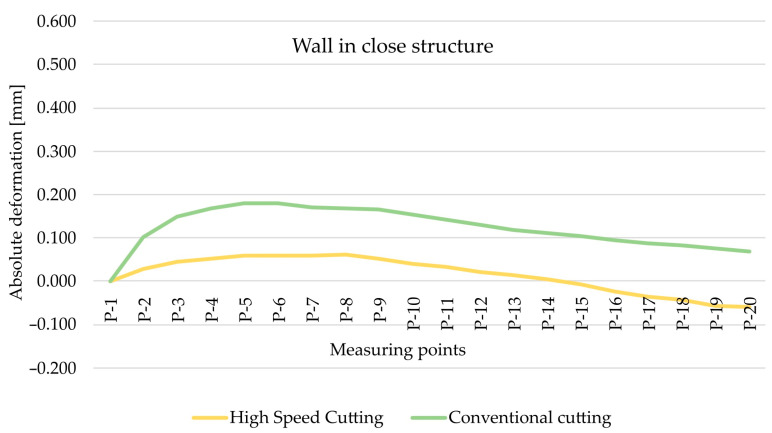
The comparison of absolute deformation for individual measurement points depending on the cutting type for the element with walls arranged in a closed structure made of the EN AW-7075 T651 aluminium alloy.

**Figure 11:**
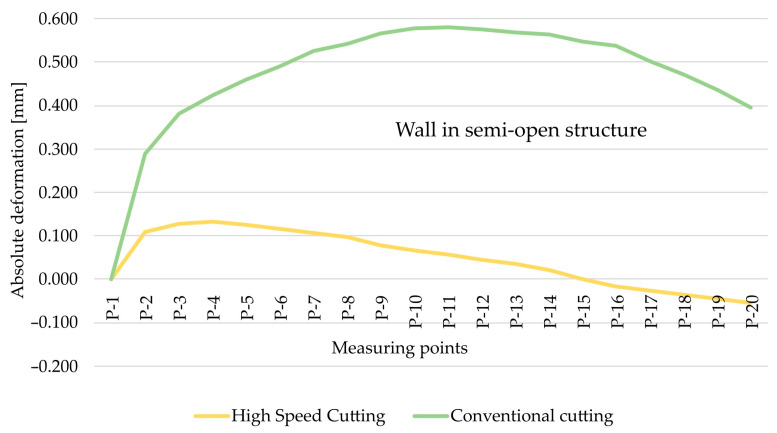
The comparison of absolute deformation for individual measurement points depending on the cutting type for the element with walls arranged a semi-open structure made of the EN AW-7075 T651 aluminium alloy.

As part of the next step, the type of finishing of the EN AW-7075 T651 was analysed. Figure 10 and Figure 11 show the comparison of absolute deformations for individual measurement points depending on the cutting type for elements with walls arranged in a semi-open and closed structure, respectively. Based on the obtained results, irrespective of the geometry of the elements, higher absolute deformation values were recorded after conventional cutting was performed with lower rotational speed *n* compared with high speed cutting.

**Figure 12:**
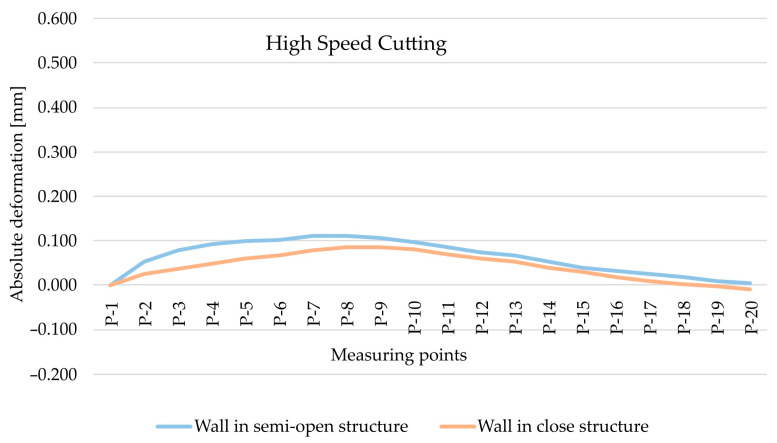
The comparison of absolute deformation for individual measurement points depending on the geometry of the thin-walled sample for HSC and for the EN AW-6082 T651 aluminium alloy.

**Figure 13:**
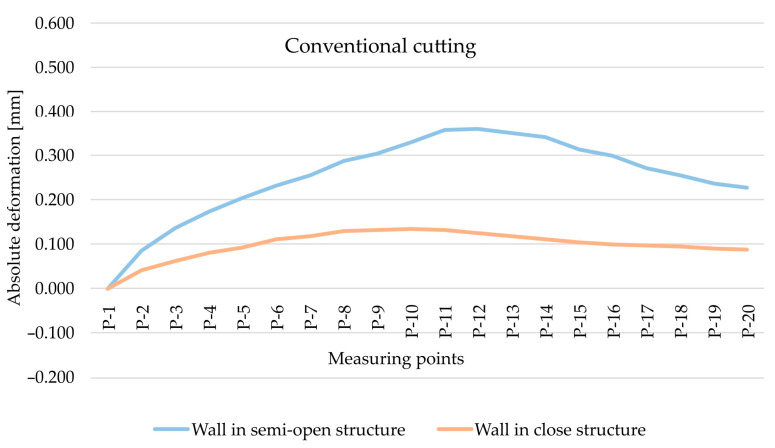
The comparison of absolute deformation for individual measurement points depending on the geometry of the thin-walled sample for conventional cutting and for the EN AW-6082 T651 aluminium alloy.

The study continued with the analysis of the results obtained for the EN AW-6082 T651 alloy and the comparison of absolute deformation values depending on the geometry of the thin-walled sample for individual measurement points, taking into account two cutting types, i.e., high speed cutting (Figure 12) and conventional cutting (Figure 13). Based on the obtained results, it has been confirmed that higher absolute deformation values were obtained for walls arranged in a semi-open structure, which results from the lower rigidity of this sample in relation to the closed-structure sample.

**Figure 14:**
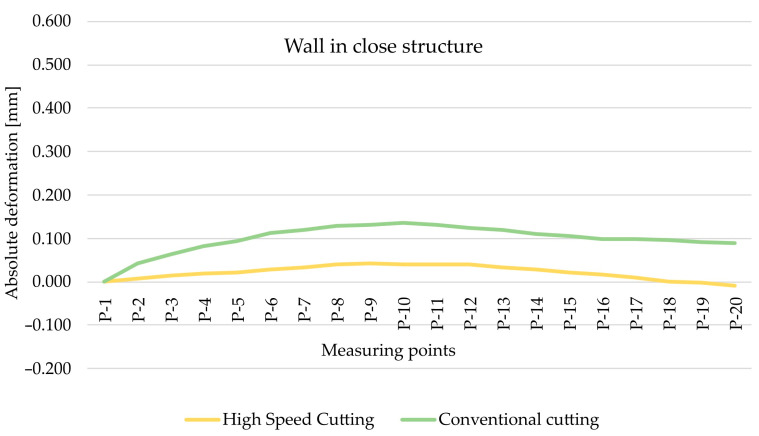
The comparison of absolute deformation for individual measurement points depending on the cutting type for the element with walls arranged in a closed structure made of the EN AW-6082 T651 aluminium alloy.

**Figure 15:**
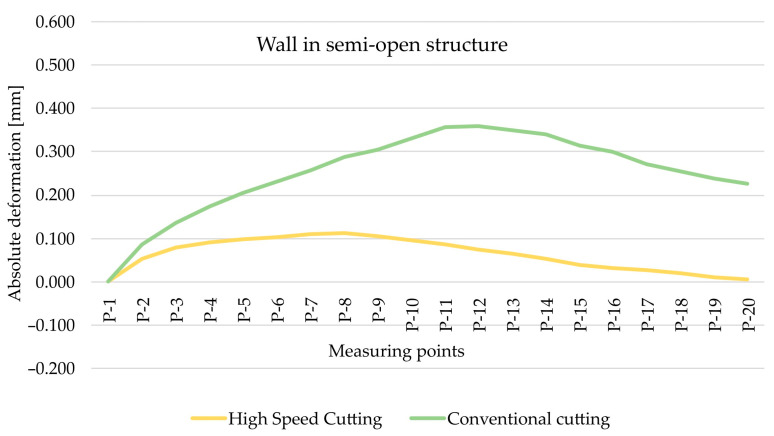
The comparison of absolute deformation for individual measurement points depending on the cutting type for the element with walls arranged in a semi-open structure made of the EN AW-6082 T651 aluminium alloy.

As regards the EN AW-6082 T651 alloy, the cutting method was also analysed. Figure 14 and Figure 15 show the comparison of absolute deformations for individual measurement points depending on the cutting type for elements with walls arranged in a semi-open and closed structure, respectively. Given this alloy, it was conclusively found that, notwithstanding the geometry of the elements, higher absolute deformation values were obtained after conventional cutting. This is consistent with the results obtained for the EN AW-7075 T651 alloy.

**Figure 16:**
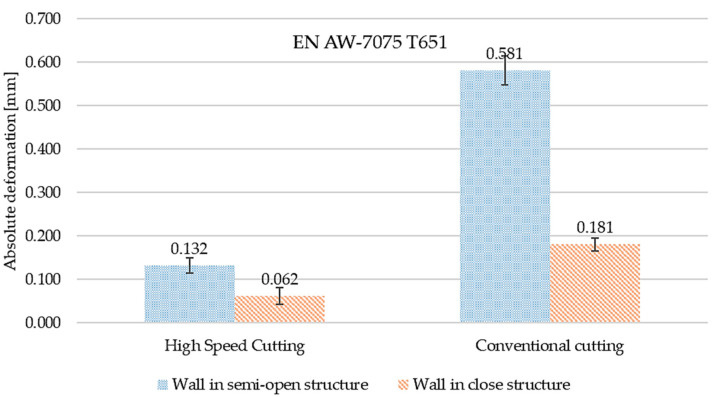
The comparison of maximum absolute deformation values depending on finishing machining and the type of structure for the EN AW-7075 T651 aluminium alloy.

**Figure 17:**
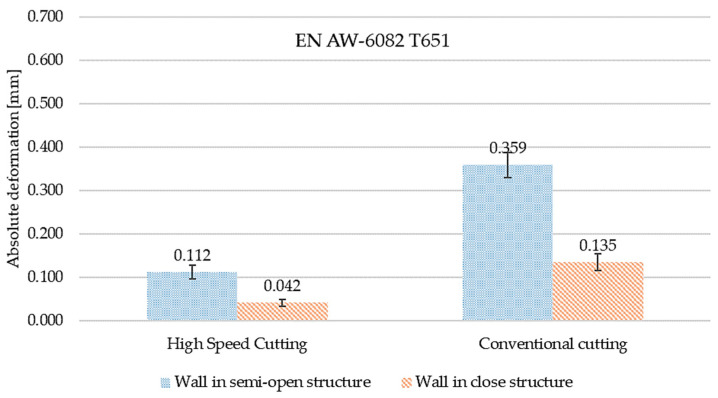
The comparison of maximum absolute deformation values depending on finishing machining and the type of structure for the EN AW-6082 T651 aluminium alloy.

Figure 16 and Figure 17 present the comparisons of maximum absolute deformation values depending on finishing and the type of structure for EN AW-7075 T651, as well as EN AW-6082 T651 aluminium alloys. Comparing the obtained results, it was found that in the case of the EN AW-7075 T651 alloy for the semi-open structure, the absolute deformation was about 440% higher for conventional machining compared to high speed cutting. In the close structure, a similar relationship was found, with the difference being over 290% (also with respect to the HSC). Comparing the results from close and semi-open structures, for HSC, the absolute deformation was almost 50% smaller for the close structure, and for conventional machining, the difference was over 30% (in relation to the open structure). Similar considerations were made for the EN AW-6082 T651 alloy, for which the same relationships were observed. In the semi-open and close structures, the absolute deformations were greater than about 320% for conventional machining compared to high speed cutting. In addition, for HSC and conventional machining, the absolute deformation in the close structure was about 40% lower in relation to the open structure.

**Figure 18:**
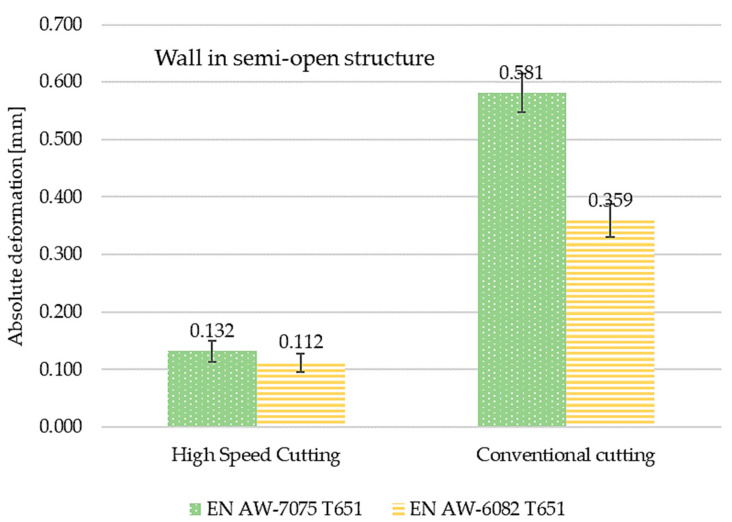
The comparisons of maximum absolute deformation values depending on finishing machining and material used for semi-open structures.

**Figure 19:**
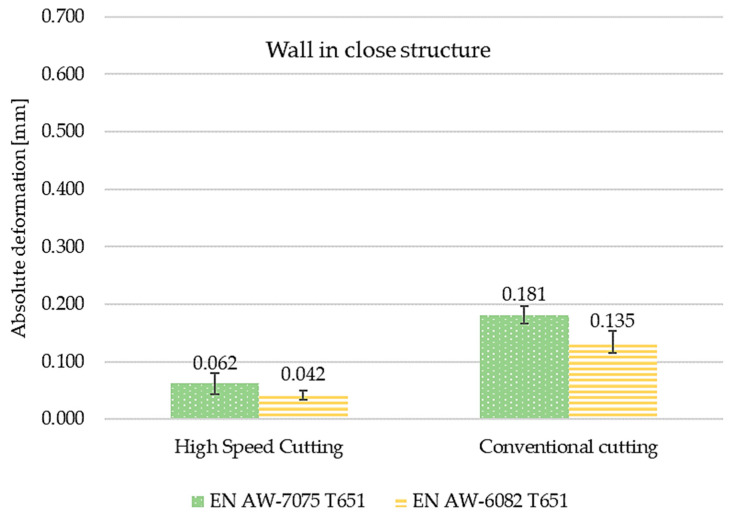
The comparisons of maximum absolute deformation values depending on finishing machining and materials used for closed structures.

The comparisons of maximum absolute deformation values depending on finishing machining and material used for semi-open and close structures are presented in Figure 18 and Figure 19, respectively. Considerations were also made for the tested materials. In each case, higher values of the maximum absolute deformation were obtained for the EN AW-7075 T651 aluminium alloy. For the semi-open structure, the absolute deformations for the EN AW-7075 T651 alloy were higher by about 120% after high speed cutting and by 160% after conventional machining. In the case of a closed structure, the differences were 150% and 135%, respectively. The reference was the absolute deformation recorded for the EN AW-6082 T651 material.

With the aim of expanding upon the present studies, it is recommended that an analysis should be performed with respect to optimising the layout of the stiffening walls.

## 4. Discussion

The conducted tests showed a significant influence of the applied technological parameters on the deformation values of thin-walled elements. Machining with parameters corresponding to HSC causes smaller deformations than after conventional machining. This is due to the fact that high speed cutting generates lower values of post-machining stresses or because the nature of these stresses is different than those observed in conventional machining. Lower stress values may be the effect of reducing cutting forces at cutting speeds corresponding to HSC, as presented in [51]. The properties of the machined material also have a significant impact on the deformation. For the EN AW-6082 T651 aluminium alloy, which is characterised by greater plasticity and lower cutting resistance, lower deformation values were observed, which is also, among others, related to the reduced cutting forces in relation to the EN AW-7075 T651 aluminium alloy.

The reduction in the deformation of thin-walled structures as a result of the use of stiffening walls is an expected and assumed effect; however, the conducted tests allowed for a quantitative comparison of the deformation values of two types of structures, i.e., close and semi-open ones. The obtained results differ depending on the material and the machining parameters used, while it was found that the maximum deformation values of the close structure for the EN AW-7075 T651 alloy are approximately 2 times lower in the case of high speed cutting, and more than 3 times lower in the case of conventional finishing than for semi-open structures. For the EN AW-6082 T651 aluminium alloy, these differences are slightly smaller. The problem that should be further analyzed is the approximation of the obtained results for the real object, which would allow for the optimization of the arrangement of its stiffening walls.

From a practical point of view, it is possible to reduce the post-machining deformations of the manufactured thin-walled elements of real aircraft structures by using properly arranged stiffening ribs, but it is also important to select the appropriate machining technology. The use of high-speed cutting, in addition to reducing the aforementioned deformations, allows an increase in the efficiency of machining.

## 5. Conclusions

Based on the test results, it can be concluded that the deformations of the analysed thin-walled structures depend both on their geometry and the residual stresses of the semi-finished product and post-machining stresses. The use of stiffening ribs brings an obvious effect that increases the rigidity of the structure and thus reduces deformations, but at the same time, it increases its mass. An important problem that requires further analyses is, therefore, the optimization of the arrangement of the stiffening ribs, allowing for an increase in stiffness with an acceptable increase in weight. During the tests, it was found that the deformations of the tested structures depend on the mechanical properties of the workpiece material and the cutting parameters used:

- Lower post-machining deformation values were obtained for the EN AW-6082 T651 alloy, which, compared to the EN AW-7075 T651 alloy, is characterised by greater plasticity and lower cutting resistance, and it translates into lower cutting forces and post-machining stresses.
- Regardless of the component geometry, higher absolute deformations were observed after conventional machining, which means that the use of high-speed HSC machining has positive effects in terms of minimizing the deformation of thin-walled components. This is probably due to the lower values of cutting forces in HSC machining.

In the next step, the authors will plan to focus on the study of residual stresses and to connect them with the resulting post-machining deformations.

## Figures and Tables

**Table 1:** The chemical composition of EN AW-7075 T651 and EN AW-6082 T651 aluminium alloys (own elaboration, based on [46]).

Content of Alloy Elements—EN AW-7075 T651 (%)										
Mg	Mn	Fe	Si	Cu	Zn	Cr	Ti	Zr + Ti	Other	Al
2.10–2.90	≤0.30	≤0.50	≤0.40	1.20–2.00	5.10–6.10	0.18–0.28	≤0.20	≤0.25	≤0.05	Rest
**Content of Alloy Elements—EN AW-6082 T651 (%)**										
Mg	Mn	Fe	Si	Cu	Zn	Cr	Ti	Zr + Ti	Other	Al
0.60–1.20	0.40–1.00	≤0.50	0.70–1.30	≤0.10	≤0.20	≤0.25	≤0.10	–	≤0.05	Rest

**Table 3:** The technological parameters of finishing machining.

Technological Parameters	Strategy	
	Conventional Cutting	High Speed Cutting
**Depth of cut** ***a_p_* (mm)**	0.2	0.2
**Cutting speed** ***v_c_* (m/min)**	200	900
**Feed per tooth** ***f_z_* (mm/tooth)**	0.02	0.02

## Data Availability

Data sharing not applicable.
